# Patient and service-level factors affecting length of inpatient stay in an acute mental health service: a retrospective case cohort study

**DOI:** 10.1186/s12888-020-02846-z

**Published:** 2020-09-07

**Authors:** Neil Crossley, Brian Sweeney

**Affiliations:** 1 Irwell Unit, Fairfield General Hospital, Rochdale Old Road, Bury, BL9 7TD UK; 2 Trafford General Hospital, Moorside Road, Davyhulme, Manchester, M41 5SL UK

**Keywords:** Psychiatry, Length, Stay, Inpatient, Factors, Mental, Health, Service, Patient

## Abstract

**Background:**

The NHS Mental Health Implementation Plan aims to reduce length of inpatient psychiatric stays to a maximum of 32 days, yet provides little guidance on how to achieve this.

Previous studies have attempted to analyse factors influencing length of stay in mental health units, focussing mostly on patient factors. These models fail to sufficiently explain the variation in duration of inpatient stay. We assess how the type of service delivered by a trust, in addition to patient factors, influences length of stay.

**Methods:**

We conducted a retrospective case cohort study in a large inner-city NHS mental health trust for all admissions in a 1 month period. Data was gathered from electronic notes of 105 patients. Descriptive univariate and bivariate analyses were conducted on the data, with multiple regression analysis conducted on statistically significant data.

**Results:**

Short-stay assessment ward admission significantly reduced length of stay. Patients under outpatients or under care co-ordination, admitted through Mental Health Act assessment and formally detained all had longer length of stay. Out of area admissions, locum Consultant care, changing Responsible Clinician and ward transfers all led to longer length of stay. Factors indicating more severe illness such as increased observation level and polypharmacy, as well as diagnoses of psychosis or bipolar disorder were associated with longer duration of stay. Discharges requiring referral to accommodation or rehabilitation led to longer stays. The most significant factors that influenced length of stay were higher observation levels, diagnosis of psychotic illness or bipolar, and discharge to rehabilitation placement.

The final model, taking into account all these factors, was able to account for 59.6% of the variability in length of stay.

**Conclusions:**

The study backs up existing literature which shows patient-factors have an influence on length of stay. The study also demonstrates that service-level factors have an impact on the duration of stay. This data may be used to inform further studies which may aid provision of inpatient and community services in the future.

## Background

Mental health policy in the UK is focussed on community-based care and reducing inpatient admissions [1]. Alongside this vision, the number of NHS inpatient mental health beds was reduced by 72.1% between 1987 and 2017, but the number of patients requiring secondary care services has continued to grow [2]. The NHS Mental Health Implementation Plan sets a target of reducing length of stay to a maximum of 32 days, putting the onus on Trusts to develop ways of achieving this [3]. Concerns that brief admissions may lead to a pattern of recurrent admissions do not seem to be supported by the evidence, and therefore provide a cost-effective model of service provision that will satisfy the goals of the long term plan [4].

As part of the NHS Long Term Plan, funding is being made available to achieve this goal, but Trusts must develop an understanding of what factors influence length of stay before implementing service change. Recent studies have looked at patient factors, demographics, diagnosis and severity in order to predict length of inpatient stay, with varying and sometimes conflicting results [5–7].

Attempts to develop an accurate model to predict length of stay have yielded poor results due to the complexity of mental health admissions [8]. Patient factors have not fully explained variations in length of stay, suggesting that other factors exert an influence. We hypothesise that factors associated with how mental health services are delivered have an impact on length of stay, potentially explaining the variation in length of stay between UK trusts [9]. There is a lack of studies addressing service factors such as the type of ward, use of locum Consultant, change in Consultant and ward transfers.

Our study aims to address this area, while also gathering more evidence of the patient-level factors to develop a better understanding of factors influencing length of stay. This information may be powerful to help trusts develop operational procedures and design services to minimise length of stay.

### Aims of the study

The aim of the study is to identify which patient and service level factors influence length of stay for general adult psychiatric inpatient admissions in a busy urban mental health NHS Trust.

## Methods

### Current setting

Our study is based in an inner-city Mental Health Trust in Manchester, United Kingdom, supporting a population of around 500,000. 66.6% of the population is White British, compared to the UK average of 85.4%, with Asian or Asian British representing the highest proportion of the black and minority ethnic population [10]. There are areas of significant social deprivation, and a higher proportion of social housing (31.6% vs 17.7%) and unemployed households (35.8% vs 33.3%) compared to the national average [10]. The area has higher prevalence of severe and enduring mental illness than the national average (1.29% vs 0.96%), a greater proportion of population in contact with mental health services, as well as higher rates of inpatient admissions, detention under the Mental Health Act and proportion of service users in hospital [11]. The prevalence of psychosis in all 3 CCGs covered by the trust is within the top ten highest in the country [11]. The trust expects to provide care for around 53,000 service users per year.

The Trust provides inpatient care spread across two sites comprising three male acute wards, two female acute wards, one mixed-gender acute ward, one female intensive care unit, one male intensive care unit and one 72-h assessment ward, a total of around 160 beds. The trust provides liaison services to three large emergency departments. Community services comprise Community Mental Health Teams (CMHT), Early Intervention Services (EIS), Assertive Outreach Team, Review Team, Home Treatment Team (HTT) and Secondary Care Psychology services.

### Study design

We used a retrospective observational study design which enabled us to identify a cohort of patients and follow them through the inpatient journey until the point of discharge using data extraction from the trust’s electronic patient records system. All consecutive patients admitted to Manchester general adult inpatient beds in May 2017 were identified for the study. Data was gathered for the 6 weeks prior to their admission, and throughout their admission until point of discharge until the cut-off point of 1st January 2018. Those discharged after the cut-off point were not included in the study as their data was incomplete.

Data were gathered on patient demographic factors; services provided in the 6 weeks prior to admission; location, time and mode of admission assessment; patient factors during admission including diagnosis and increased observation levels; service factors during admission including out of area admission, inter-ward transfers, change of Consultant, Locum Consultant involvement, polypharmacy, type of ward, involvement of patient flow team (through identification of delayed transfer of care [DTOC]) and detention under Mental Health Act; and discharge service factors including referral to community teams and accommodation status at discharge. Data on diagnosis at discharge was obtained from final ward round entry or discharge summary, and was often not recorded as per ICD-10 criteria and lacked information on comorbidity. Therefore the researchers pragmatically classed diagnoses into broad categories of depression, psychosis, bipolar disorder, personality disorder, substance misuse disorder and other (which included ‘malingering’, ‘no mental disorder’, etc).

Data was extracted from electronic records to ensure anonymity. Permission to obtain data was obtained from the Trust’s Clinical Audit Department. As this study constitutes an audit or service evaluation, no ethical approval was required from the NHS Research Ethics Committee. Data was transcribed onto an Excel spreadsheet and subsequently analysed using IBM SPSS version 26.

### Data analysis

Initial descriptive and univariate analyses were carried out on the data. Bivariate analyses were carried out using Length of Stay (LOS) as the dependent variable. LOS was calculated as number of days between admission and discharge, and was classed as 0 for some patients who were discharged within 24 h. LOS is typically a skewed distribution due to a small proportion of patients having prolonged inpatient admission, therefore non-parametric tests were used to analyse significant effects on LOS. Categorical data was analysed using the Mann-Whitney test where only two groups exist, and the Kruskal-Wallis test where several groups exist. Continuous data was analysed using Spearman’s Rank correlation coefficient. *P*-value of ≤0.05 was considered to be significant.

Multiple regression analysis was then used with the independent variables which had demonstrated statistical significance from bivariate analysis to generate a model of factors influencing the LOS. This technique was chosen to avoid the use of an arbitrary cut-off between short- and long-stay patients which is necessary for other forms of statistical analysis.

## Results

### Sample

106 patients were admitted during May 2017. One patient’s records could not be accessed due to an incorrect patient identifier number and therefore was not included in the study. A further 8 patients remained an inpatient at the point of data collection in January 2018 and therefore were not included in the study. The remaining 97 patients were included in the analysis.

The mean age of the sample was 36, with 67% being male. Psychosis (30%) was the most common reason for admission, followed by personality disorder (21%), depression (8%) and bipolar disorder (8%). While the majority of patients were admitted from some form of accommodation, 10% were street homeless. Table 1 shows the sample demographics.

**Table 1:** Sample demographics

	n (%)
**Gender**	
Male	65 (67)
Female	32 (33)
**Age**	
16–24	13 (13)
25–34	42 (43)
35–44	17 (18)
45–54	15 (16)
55–64	8 (8)
65+	2 (2)
**Diagnosis at discharge**	
Depression	6 (6)
Psychosis	31 (32)
Bipolar Affective Disorder	8 (8)
Personality Disorder	20 (21)
Substance Misuse Disorders	11 (11)
Anxiety Disorder	3 (3)
Other	8 (8)
Unknown (not documented)	10 (10)
**Medication**	
No medication	6 (6)
One medication	28 (29)
Polypharmacy	53 (55)
Unknown	10 (10)

### Descriptive and bivariate statistics

The median LOS was 22 days, with a mean LOS of 36.1 days, demonstrating a positive skew. A number of patients had inpatient stays of less than 24 h, with the longest admission being 226 days.

Patients under HTT, CMHT or other community teams in the 6 weeks prior to admission did not have longer LOS. However, those allocated to a care coordinator in the community (*p* = 0.033) or who were under secondary care outpatients (*p* = 0.034) had significantly longer admissions. Higher frequency of contacts from HTT was associated with a reduced LOS, but this was not statistically significant. Readmissions, A + E presentations and Mental Health Act assessments within the last 6 weeks were not shown to have an impact on LOS.

The day, timing and location of admission assessment did not have an impact on LOS. Admission via a Mental Health Act assessment was associated with significantly longer LOS (*p* = 0.005), with significantly longer admissions for those formally detained under Section 2 or 3 (*p* ≤ 0.001).

Admission to and discharge from the short-stay assessment ward was associated with significantly reduced LOS (*p* ≤ 0.001), but there was no difference between people subsequently transferred to an acute ward from the assessment ward.

Out of area admissions (*p* = 0.002), involvement of locum Consultant (*p* ≤ 0.001), increased observation level (p ≤ 0.001) and polypharmacy (*p* = 0.031) were all associated with longer admissions. Increasing number of ward transfers (*p* ≤ 0.001) and Consultant changes (*p* ≤ 0.001) were shown to be associated with increased LOS. Patients requiring rehabilitation (*p* = 0.04) or referral to accommodation at discharge (*p* = 0.018) had significantly longer inpatient stays.

In terms of patient factors, diagnosis of psychosis or bipolar disorder (*p* ≤ 0.001) was associated with longer admissions. Other diagnoses had no significant impact. Gender, age and accommodation status was not shown to impact LOS.

The results of the analysis are shown in Table 2 (categorical data) and Table 3 (continuous data).

**Table 2:** Analysis of factors influencing LOS (categorical data)

			Length of Stay (days)					*p* value
	Items	n	Mean	Median	IQR	Min	Max	
Length of Stay		97	36.10	22	40	0	226	
Gender	M	65	35.43	17	38	0	226	0.326
	F	32	37.44	28	47	1	147	
Accomodation	Independent	78	33.71	19	41	0	147	0.449
	Hotel	2	22.00	22	–	3	41	
	Supported	3	36.67	28	–	28	54	
	NFA	10	61.60	27	91	2	226	
	Family	2	3.50	3.50	–	2	5	
	Prison	1	40	–	–	–	–	
	Nursing	1	55	–	–	–	–	
Previous admission	Y	9	35.67	16	54	1	140	0.852
	N	88	36.14	22	42	0	226	
Previous A + E Presentation	Y	28	36.89	21	56	0	147	0.946
	N	69	35.77	22	33	1	226	
Previous MHA Assessment	Y	7	60.57	55	74	1	147	0.120
	N	90	34.19	18	36	0	226	
HTT Referral	Y	34	36.91	23.50	51	1	147	0.889
	N	63	35.65	19	40	0	226	
Under HTT	Y	35	36.06	22	48	1	147	0.723
	N	60	36.82	20.50	40	0	226	
HTT Location	A	10	28.10	6.50	45	1	123	0.364
	B	17	36.06	27	42	4	140	
	C	10	41.20	20	79	1	147	
HTT Medical Review	Y	11	22.55	7	21	1	140	0.077
	N	86	37.83	23	48	0	226	
Under CMHT	Y	30	44.87	27.50	59	1	147	0.092
	N	67	32.16	16	35	0	226	
CMHT Location	A	1	61	–	–	–	–	0.664
	B	7	49.43	35	82	2	124	
	C	6	24.00	27.50	34	3	41	
	D	9	59.33	28	93	14	140	
	E	1	25	–	–	–	–	
	F	6	39.33	16	77	1	147	
Allocated CC	Y	27	48.44	28	69	1	147	0.033
	N	70	31.33	16	24	0	226	
Under RC	Y	56	43.70	27.50	54	1	226	0.034
	N	41	25.71	15	26	0	212	
Under other community team	Y	33	38.45	22	36	1	226	0.973
	N	64	34.88	21.50	46	0	147	
Day of Admission	Mon	13	17.62	8	18	1	80	0.115
	Tue	15	28.53	16	21	1	226	
	Wed	16	36.00	19	47	1	131	
	Thu	18	51.06	40.50	65	2	147	
	Fri	14	37.14	29.50	21	0	124	
	Sat	9	32.33	25	41	2	88	
	Sun	12	44.83	16.50	88	1	212	
OOH	Y	63	38.52	22	47	0	212	0.391
	N	34	31.59	16	36	1	226	
MHA Assessment	Y	56	43.04	28.50	47	0	212	0.005
	N	41	26.61	14	23	1	226	
Route of admission	Community	20	36.50	19.50	31	1	226	0.286
	A + E	62	40.44	27	47	0	212	
	Liaison	11	15.91	6	14	2	57	
	Criminal Justice System	3	21.33	16	–	8	40	
	HTT	1	25	–	–	–	–	
Legal Status	Inf	53	19.85	10	22	0	147	≤0.001
	S2	40	49.43	38.50	44	2	121	
	S3	3	82.00	83	–	39	124	
	CTO recall	1						
Ax ward admission	Y	56	24.86	7.50	30	0	212	≤0.001
	N	41	51.44	31	55	5	226	
Ax ward discharge	Y	31	5.81	4	4	1	48	≤0.001
	N	66	50.32	32	43	0	226	
Acute Ward	A	6	33.33	32	15	17	56	0.301
	B	4	23.25	24.50	9	16	28	
	C	3	95.00	31	–	28	226	
	D	7	53.14	40	61	16	123	
	E	3	96.33	110	–	55	124	
	F (PICU)	7	42.14	19	39	8	140	
	G (PICU)	2	67.50	67.50	–	39	96	
	H	3	77.33	80	–	5	147	
Out of area admission	Y	31	46.16	37	47	0	131	0.002
	N	66	31.36	16	30	1	226	
Locum Consultant	Y	28	49.68	34	38	8	140	≤0.001
	N	69	30.58	14	32	0	226	
Increased obs level	Y	24	68.08	47.50	67	8	226	≤0.001
	N	73	25.58	16	31	0	131	
Discharge Diagnosis	Depression	8	22.88	6	17	2	131	≤0.001
	Psychosis	29	62.62	40	66	7	226	
	Bipolar	8	53.88	44.50	54	25	96	
	Personality disorder	20	20.50	6	23	1	147	
	Substance misuse	11	20.09	16	35	2	57	
	Other	21	20.95	10	29	0	111	
Medication	Nil	6	23.17	20	41	2	57	0.031
	Monotherapy	28	20.64	9	22	2	124	
	Polypharmacy	53	48.68	29	61	1	226	
Identified DTOC	Y	2	127.00	127.00	–	28	226	0.144
	N	95	34.18	19	40	0	212	
Discharge destination	Home	75	30.53	17	33	0	131	0.04
	Supported	2	28.00	28.00	0	28	28	
	Family	5	26.40	16	51	2	63	
	NFA	6	16.50	8	36	2	40	
	Rehab	2	183.00	183.00	–	140	226	
	Other	7	79.71	55	114	2	212	
Discharge Team	CMHT	26	45.27	28	62	1	131	0.069
	HTT	44	25.30	16	30	1	111	0.106
	Other	17	29.24	17	34	1	110	0.917
Referral to accommodation	Y	2	183.00	183	–	–	–	0.018
	N	95	33.00	19	40	0	212

**Table 3:** Analysis of factors influencing LOS (continuous data)

	r	*p* value
Age	0.084	0.414
HTT Contacts	−0.116	0.495
CMHT Contacts	0.095	0.617
Ward Transfers	0.523	≤0.01
Consultant Changes	0.592	≤0.01

### Multiple regression analysis

Statistically significant factors from bivariate analyses were used for multiple regression analysis. The results are shown in Table 4. Factors were added in a stepwise fashion based on their level of statistical significance. First order linear auto-correlation was ruled out using the Durbin-Watson calculation (d = 1.82). Multicollinearity was ruled out as tolerance was > 10. The model showed a statistically significant ability to predict length of stay (F statistic = 8.30, *p* ≤ 0.001). The model accounted for 59.6% of the variability displayed in LOS.

**Table 4:** Multiple regression analysis; R^2^ = .596, *p ≤ 0.05, **p ≤ 0.01

	b	SE b	*β*
(Constant)	19.80	17.87	
Allocated care coordinator (6/52 prior to admission)	4.81	9.21	.048
Under RC (6/52 prior to admission)	7.73	8.66	.083
Admitted under MHA	9.85	7.37	.106
Admitted to Ax Ward	17.07	9.41	−.180
Elevated observation levels	18.34	8.90	.179*
Referral to accommodation on discharge	72.75	28.42	.238*
Out of area admission	20.19	10.34	.189
Locum RC	−19.21	10.20	−.196
Diagnosis at discharge	−6.70	2.33	−.236**
Medication regime	8.16	6.12	.111
Discharge destination	7.91	2.60	.254**
Consultant changes	9.16	6.50	.204
Ward transfers	2.56	7.74	.046

The most statistically significant factors impacting length of stay were elevated observation levels (increasing admission up to 18 days), diagnosis at discharge (personality disorders, substance-misuse disorders and ‘other’ disorders reduced length of stay compared to affective and psychotic illnesses) and discharge destination (rehabilitation) which increased length of stay by 72 days.

## Discussion and conclusions

### Discussion

We performed a study of a large urban NHS Trust aiming to identify which patient and service factors influenced length of stay for general adult acute inpatients. Patient factors shown to increase length of stay included diagnosis of psychotic illness, necessity for increased observation levels and detention under the Mental Health Act.

Service level factors shown to influence length of admission are the allocation of a care coordinator or secondary care outpatient appointments prior to admission, out of area admissions, management from a locum Consultant, Consultant changes, ward transfers and polypharmacy. The short-stay assessment ward was shown to have a positive impact in terms of reducing length of stay. Discharge planning, specifically around referral to accommodation and rehabilitation, was a significant delaying step, prolonging inpatient admission.

Other studies have shown similar impact of observation levels and seclusion on length of stay [12]. Factors which infer a greater degree of severity of illness, such as increased observation levels, polypharmacy and detention under Mental Health Act were all shown to increase length of stay, however rating scales for severity of mental illness were not available.

Our results supported previous studies that have demonstrated a significant impact of psychosis on length of stay and a negligible impact of substance use disorders and personality disorders on length of stay [12–14]. While international studies often demonstrate reduced length of stay in detained patients [6], our findings support UK studies that show the use of the Mental Health Act is related to longer admissions [15], perhaps suggesting compulsory detention is used differently in the NHS.

Unlike other studies, we did not find an association between homelessness and length of stay [15, 16] but we did find a similarly significant impact of the requirement for referral to accommodation on discharge. In keeping with the conflicting data surrounding age and length of stay, we were unable to demonstrate any significant association [14].

Our study looked specifically into factors associated with services provided by the Trust. The short stay assessment ward was demonstrated to significantly reduce length of stay, supporting results from a similar service in the UK [17]. Additionally, subsequent transfer from the assessment ward to an acute ward did not significantly prolong length of stay.

Out of area admissions have already been shown to increase length of stay [18] and our findings support this conclusion. Additionally, within-Trust continuity of care appears to be an important factor in length of stay. We have demonstrated that transfers between wards and changes in Consultant can have a significant impact on length of stay.

### Limitations

The sample size for this study was small, relating to one NHS Trust and has follow-up data covering less than 1 year. Some patients who remained an inpatient at the time of data collection could not be included in the study, which may have impacted on the results. While these patients are likely to be outliers, they could have had a considerable impact on the results.

It is worth noting that the study takes place in one particular trust, which as described earlier has a high degree of illness and social deprivation. It may be that due to these factors, the results are not generalisable to other areas. While the authors feel the patient cohort may be representative of other similar urban areas in the UK, the provision of services is dependent on Trust with a wide variation in quality. Services provided by Trusts are dependent on several other factors not considered here including quality of staff, staffing levels, physical environment, make-up of the multi-disciplinary team and a variety of other factors which are beyond the reach of this study. Therefore, any conclusions drawn from this study should be interpreted with caution when considering applicability to other services. The authors suggest this study could be used as a proof of concept for a larger study that is not confined to one particular trust, which could therefore be more generalisable.

Other studies have looked at both psychopathology and ICD-10 diagnostic criteria as factors for influencing length of stay [7, 8], however due to poor and inconsistent documentation on the electronic patient record the researchers were unable to be as stringent in their data gathering for diagnosis, instead relying on broader categories. In addition, due to variations in documentation practice in the service, it was not possible to ascertain comorbidities with any confidence. It is likely that a high proportion of patients may have had several diagnoses which have not been captured by this study. While severity of illness may be inferred by some of the statistics (for example the use of increased observation levels, polypharmacy and detention under Mental Health Act may suggest more severe illness), firm conclusions cannot be drawn from this. There was no recorded data using rating scales to define severity of illness, and this may have had a significant impact on length of inpatient stay. Further study on the impact of comorbidities and degree of illness severity to confirm our tentative findings would be useful.

Our data takes into account the provision of a short stay assessment ward which is included in the analysis. Other such services are seldom found in UK NHS Trusts and therefore data relating to the assessment ward and overall data may not be transferrable to other settings. While we have assessed its impact on this admission, studies have demonstrated a slight increase in risk of readmission, which has not been considered in this study [17].

### Conclusions

Our study reinforces several of the findings from previous studies regarding factors that influence length of stay for acute psychiatric patients. Length of stay is multifactoral and will likely depend on a combination of several factors including patient- and service-level factors. We have demonstrated that patient factors including diagnosis, need for increased observations and multiple medications have significant impact. Our study supports the previous findings that accommodation and support post-discharge is often a stumbling block for discharging patients who are otherwise ready for discharge. The results suggest that discharge planning should commence immediately on admission to reduce downstream delays, and interfaces with social housing and other accommodation services should be better developed.

We have also demonstrated that service-level factors play a role in length of stay. Particularly, continuity of care can be a significant factor in prolonging length of stay. We suggest that length of stay could be reduced by improving continuity of care wherever possible so that patients are seen within-Trust by consistent staff in a consistent location, with minimal transfers. The concept of continuity of care to reduce inpatient stay is in harmony with the NHS vision for reducing the number of inappropriate out of area placements and reducing length of inpatient stay.

We have demonstrated that the acute assessment ward model is effective in reducing length of stay for appropriate patients, and does not significantly prolong length of stay for those who may require a subsequent acute ward admission, however we did not assess the risk of readmission in these patients. Further research to look at readmission rates and subsequent length of stay would is necessary.

Due to the limitations of this study in terms of sample size and limited data available regarding comorbidities, the authors suggest this study be used as a proof of concept for a much larger study using larger linked datasets that are not limited to one particular trust, and therefore could be more generalisable to the NHS and healthcare as a whole. While some service-level factors were considered, as discussed above, there are myriad factors which will be in some cases universal, and in some unique per trust including outdated estates, understaffed inpatient units and reduced skill mix within under-resourced teams. It is likely that these and other service level factors not considered by this study could have a vital impact on length of stay, which is worthy of further exploration as Trusts struggle to identify how to maintain a quality service provision and reduce length of stay.

## Data Availability

The datasets used and analysed during the current study are available from the corresponding author on reasonable request.

## Abbreviations

NHS: National Health Service
CCG: Clinical Commissioning Group
EIS: Early Intervention Service
ICD-10: International Classification of Diseases, Volume 10
LOS: Length of Stay
NFA: No fixed abode
A + E: Accident and emergency
MHA: Mental Health Act
HTT: Home Treatment Team
CMHT: Community Mental Health Team
CC: Care Co-ordinator
RC: Responsible Clinician
OOH: Out of Hours
Ax Ward: Assessment Ward
Obs: Observations
DTOC: Delayed Transfer of Care
